# Fishery waste valorization: Sulfated ZrO_2_ as a heterogeneous catalyst for chitin and chitosan depolymerization

**DOI:** 10.3389/fchem.2022.1057461

**Published:** 2022-11-02

**Authors:** Valeria Pappalardo, Yassine Remadi, Laura Cipolla, Nicola Scotti, Nicoletta Ravasio, Federica Zaccheria

**Affiliations:** ^1^ CNR Institute of Chemical Sciences and Technologies “G. Natta”, Milano, Italy; ^2^ Department of Biotechnology and Biosciences, University of Milano—Bicocca, Milano, Italy

**Keywords:** chitin, chitosan, solid acids, ZrO_2_, sulfated catalyst

## Abstract

Chitin and chitosan are abundant unique sources of biologically-fixed nitrogen mainly derived from residues of the fishery productive chain. Their high potential as nitrogen-based highly added-value platform molecules is still largely unexploited and a catalytic way for their valorization would be strongly desirable within a biorefinery concept. Here we report our results obtained with a series of heterogeneous catalysts in the depolymerization of chitosan and chitin to acetylglucosamine. Copper catalysts supported on SiO_2_, SiO_2_–Al_2_O_3_, SiO_2_-ZrO_2_, ZrO_2_ and the corresponding bare oxides/mixed oxides were tested, together with a sulfated zirconia system (ZrO_2_-SO_3_H) that revealed to be extremely selective towards glucosamine, both for chitosan and chitin, thus giving pretty high yields with respect to the values reported so far (44% and 21%, respectively). The use of a heterogeneous catalyst alone, without the need of any additives or the combination with a mineral acid, makes these results remarkable.

## Introduction

Chitin is the second most abundant biopolymer besides cellulose and is the first aminosaccharide based-one. It can be extracted on large scale (10^11^ tons/year) from crustaceans shell waste, the industrial waste material of fisheries, through a well-established protocol, and it can be also obtained from insects. (Jia et al., 2014) (Hou et al., 2020) (Sagawa et al., 2019) (Mohan et al., 2022).

For these reasons, chitin and its deacetylated form, chitosan, can be considered as relevant second generation renewable raw materials, that through biorefinery processing produce added-value platform molecules, particularly when the nitrogen-containing functional group is preserved. Despite this, their applications are often limited to wastewater treatment, food industry, cosmetics, and nanomaterials and composite materials preparation. (Yan and Chen, 2015) (Gómez-López et al., 2020) (De et al., 2015) (Ahmed et al., 2020) (Jayakumar et al., 2010) (Sanandiya et al., 2020) (Deliyanni et al., 2015) (Mäki-Arvela et al., 2007) Thus, chitin and chitosan represent abundant and unique sources of biologically-fixed nitrogen nowadays still largely un-exploited.

**FIGURE 1:**
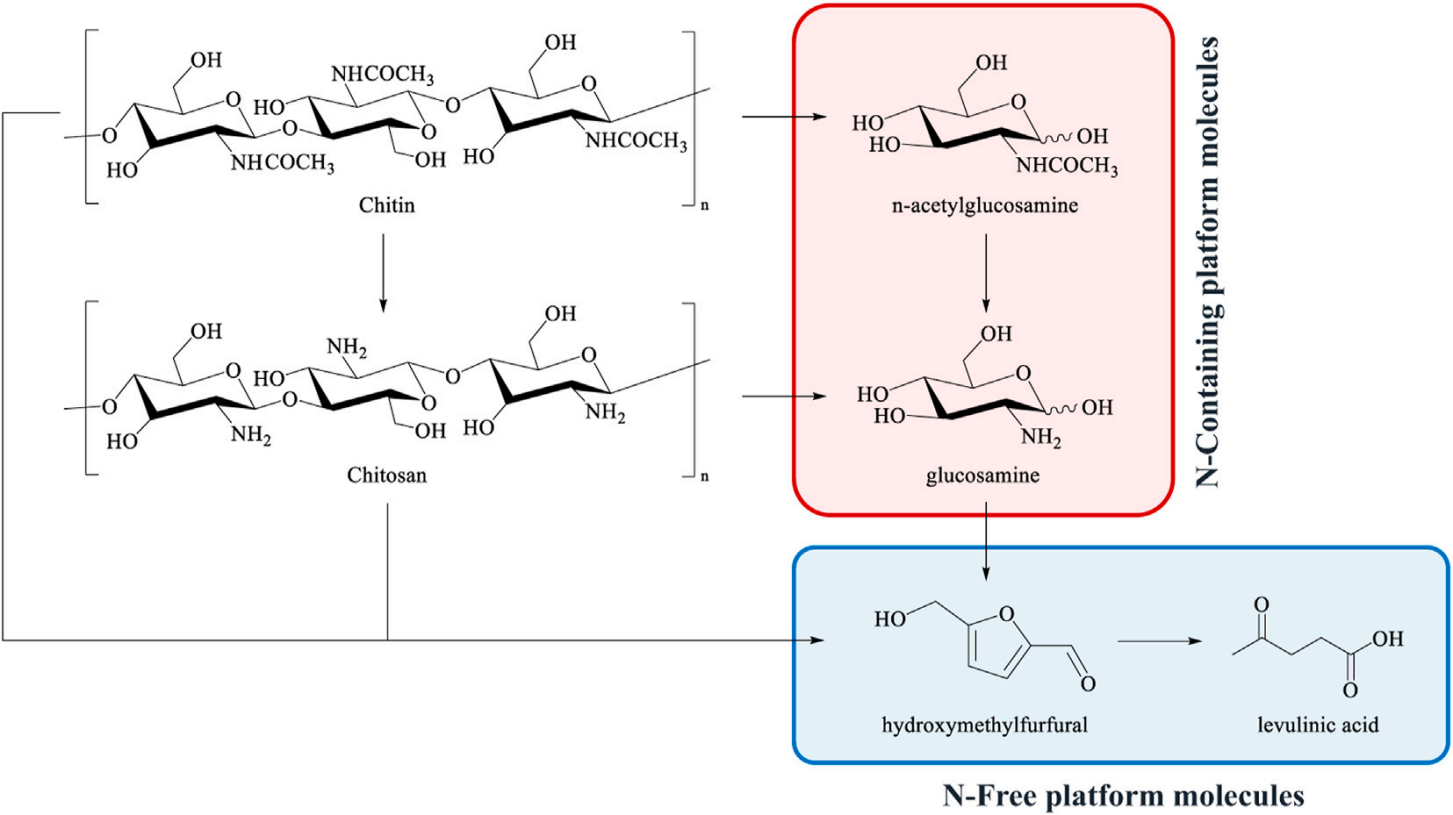
Main platform molecules derived from the valorisation of chitin and chitosan.

By applying the same approach used for cellulose and hemicellulose, chitin and chitosan can be depolymerized into their constituent monomers, namely 2-acetamido-2-deoxy-D-glucose (*N*-acetylglucosamine, GlcNAc) and 2-amino-2-deoxy-D-glucose (glucosamine, GlcN), that are the starting point for the synthesis of new and sustainable renewable molecules in which the nitrogen atom is retained (Figure 1). (Pelckmans et al., 2017) (Omari et al., 2012) (Ohmi et al., 2013) (Sadiq et al., 2018) (Osada et al., 2013) (Dai et al., 2019) (Sagawa et al., 2019) (Jia et al., 2017) (Drover et al., 2012) (Xu et al., 2022).

For example, the catalytic oxidation of GlcN affords the value-added glucosaminic acid. (Ohmi et al., 2013) GlcN can also be dehydrated into deoxyfructosazine and fructosazine, that find application as flavouring agents, but also show potential antimicrobial and pharmacological activity. (Jia et al., 2014) (Jia et al., 2017) (Bhattacherjee et al., 2016) Typically, these compounds price is around 70/mg USD and 32/mg USD by Santa Cruz Biotech-Nology, respectively. (Jia et al., 2014) (Jia et al., 2017) Analogously to 5-(hydroxymethyl)furfural (HMF) from glucose, the dehydration of GlcNAc leads to the 3-acetamido-5-acetylfuran, a promising N-containing platform molecule that provides many opportunities for accessing a wide range of products. Omari et al. (2012), Drover et al. (2012), Padovan et al. (2020), Sadiq et al. (2018), Chen et al. (2014) GlcNAc can be also converted into Chromogen I and III that recently have attracted great attention as new functional food additives and as potential therapeutics, having potent biological activities. (Osada et al., 2013) (Zheng et al., 2017) At the same time, the combination of hydrogenation and dehydration reactions leads to the 2-acetamide-2-deoxyisosorbide, an analogous of isosorbide, which is a new potential precursor to N-containing polymers. (Sagawa et al., 2019).

On the other hand, when the nitrogen atom is lost, the valorisation of chitin and chitosan produces compounds such as HMF, levulinic acid and C2–C6 polyols, similarly to what happens in the cellulose and hemicellulose deconstruction pathways (Figure 1). (Kim et al., 2018) (Hou et al., 2020) (Omari et al., 2012) (Wang et al., 2013) (Bobbink et al., 2015) (Pandit et al., 2020) (Ohmi et al., 2013) (Padovan et al., 2020) (Xu et al., 2022) (Kougioumtzis et al., 2018).

In contrast to the valorization of cellulose and hemicellulose, chitin and chitosan depolymerization studies are only at early stages, despite the similar chemical structure of these natural polymers. Even if more efforts would be needed, particularly aimed at improving selectivity and at reducing waste, the transformation of cellulose and hemicellulose can inspire chitin and chitosan exploitation, which is still very limited.

The first step for the profitable use of chitin and chitosan as feedstock for biorefineries is the identification of an effective depolymerisation protocol to obtain oligomers or the corresponding monomers. Few attempts are reported in the literature. Most of the works relies on the use of toxic and dangerous homogeneous systems, based on mineral acids (HNO_3_, H_2_SO_4_, and HCl) or free radicals, sometimes with the aid of mechanical treatments, such as ball-milling. (Harish Prashanth and Tharanathan, 2007) (Einbu and Vårum, 2008) (Zhang and Yan, 2017) (Yabushita et al., 2015) (Kobayashi et al., 2021) The use of heterogeneous catalysts is more convenient, since they are safer to handle, generally less toxic and, with respect to mineral acids, they entail lower purification costs and they do not require the neutralization of the reaction medium. Unfortunately, the processes reported are effective only after chitosan solubilization in a mineral acid, such as H_2_SO_4_ and HCl, as in the case of Amberlyts-15 (GlcN yield = 58%) (Sagawa et al., 2019), and of a glucose-derived solid acid (GlcN yield = 98.1%). (Zhang et al., 2018) On the other hand, when a solid acid catalyst is used alone (e.g., H-Mordenite), low molecular weight chitosan oligomers are obtained. (Pandit et al., 2020) Sometimes, during the reaction the loss of nitrogen atoms occurs. In these cases, the formation of the products usually derived from cellulose and hemicellulose valorisation, such as levulinic acid and HMF, is observed. (Omari et al., 2012) (Hou et al., 2020).

In this work we investigated the activity of some oxides and mixed oxides as simple heterogeneous catalysts for the selective conversion of chitosan and chitin into GlcN.

## Experimental

### Materials

All chemicals, in particular low molecular weight chitosan, chitin from shrimp shells and HPLC standards (formic acid, acetic acid), were obtained from Sigma-Aldrich, with the exception of HMF that was purchased from AVA Biochem.

ZrO_2_ (Melcat XZO1521), SiO_2_–Al_2_O_3_ 135 (Aldrich), SiO_2_ A (Merk), and pyrogenic SiO_2_ B (Aeroperl 300/30) are commercial materials respectively purchased from Mel-Chemicals, Sigma-Aldrich and Evonik. SiO_2_-ZrO_2_ containing a 4.7% nominal amount of zirconia was kindly provided by Grace Davison (Worms, Germany) Textural properties are reported in Table 1.

**TABLE 1:** Physiochemical characterization of the materials.

Sample	SSA (m^2^/g)	PV (cm^3^/g)
SiO_2_ A	460	0.82
SiO_2_ B	267	1.65
SiO_2_-Al_2_O_3_	496	0.74
SiO_2_-ZrO_2_	304	1.62
ZrO_2_	358	0.20

SSA, Specific Surface Area; PV = Pore Volume.

### Catalysts preparation

Copper catalysts were prepared according to the Chemisorption-Hydrolysis method (Zaccheria et al., 2013) (Scotti et al., 2017) by adding 10 g of support (SiO_2_ A, SiO_2_ B, and SiO_2_–Al_2_O_3_) to an aqueous [Cu(NH_3_)_4_]^2+^ solution, prepared by dropping NH_4_OH to a 25 mL of a Cu(NO_3_)_2_·3H_2_O solution until pH 9 had been reached. After 20 min under stirring, the slurry, held in an ice bath at 0°C, was diluted with 3 L of water for 2 h. The solid was separated by vacuum filtration with a Büchner funnel, washed with water, dried overnight at 110°C, and calcined in air at 350°C for 4 h.

Sulphonated ZrO_2_ catalyst was prepared according to Fărcaşiu and Li, 1995 protocol. 5 g of the oxide were soaked in 75 mL of aq. 1 M H_2_SO_4_ for 2 h, filtered, and dried overnight at 110°C. Finally, the dried material was calcined at 350°C for 4 h.

### Chitin and chitosan hydrolysis

Before reaction, the catalyst (400 mg) was placed in a glass reactor and dehydrated at 270°C in an oven (20 min in air +20 min under vacuum). The reactions were performed in a Hastelloy Parr autoclave. The reactor was loaded with 750 mg of chitin/chitosan, 50 mL of H_2_O and the dried catalyst. The autoclave was sealed, evacuated, and filled with 4 atm of N_2_. The system was heated up to the desired temperature under stirring (750 rpm). After the desired time, the autoclave was vented, and the catalyst and the insoluble residues were separated by filtration.

### Product analysis

Products were analysed by HPLC using an Agilent 1,200 Infinity LC system equipped with a Quaternary gradient pump unit and a refractive index (RID) detector. MetaCarb H Plus Column 300 × 7.8 mm (Agilent Technologies, Inc.), hyphenated with MetaCarb H Plus Guard Column 50 × 4.6 mm, was used as analytical column. MilliQ water was used as the mobile phase and the separation was carried for 2 h under the following conditions: flow rate 0.4 mL/min at 60°C, wavelength *λ* = 195 nm, RID temperature 35°C. Before analysis, the reaction mixture was filtered through a 0.45 μm microporous membrane, diluted with milliQ water at a 0,66 pV dilution factor, and injected (20 μl) with a 50 μl glass syringe (Agilent Technologies, Inc.). To calculate the GlcN yield (wt%), a linear calibration curve (r2 > 0.99) was obtained using GlcN as standard and the following equation was used:

$$Y\left(wt\%\right)=\frac{{mmol}_{GlcN}∙{MM}_{GlcN}}{{mg}_{reagent}},$$ (1)

Where mmol_GlcN_ is obtained from the HPLC analysis and mg_reagent_ refers to chitin/chitosan.

The conversion of chitin and chitosan into soluble products was calculated through total organic carbon (TOC) analysis on the liquid filtrated solutions. TOC analysis was performed using a TOC-LCSH analyser purchased from Shimadzu. Samples for TOC were all prepared at a 16 dilution factor mixing 3.125 mL of the filtered reaction mixture with milliQ water to a final volume of 50 mL. The instrument performs the analysis of the TC and IC and create the TOC value given the relationship TOC = TC—IC.

## Results and discussion

In a previous work, some of us reported that the selectivity in the hydrolysis of cellulose can be addressed by using different catalysts based on oxides and mixed oxides (e.g. SiO_2_, SiO_2_-ZrO_2_, and SiO_2_-Al_2_O_3_) and the corresponding copper-based ones. The Lewis and Brønsted acidity features of the tested materials accounted for the different distribution of the products obtained. (Mariani et al., 2014) In particular, when a CuO/SiO_2_ catalyst is used glucose is obtained in high amount. On the other hand, when ion-like copper species, as those formed over a SiO_2_-Al_2_O_3_, were present, lactic acid is the main product. With respect to the non-catalysed reaction, the bare supports have an important effect on the selectivity, giving significantly higher amounts of glucose and HMF.

**SCHEME 1:**
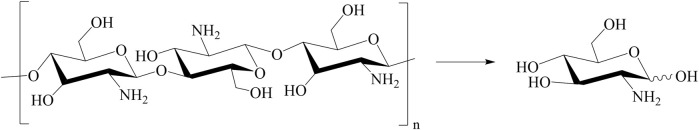
Chitosan depolymerisation to GlcN.

Based on the previous promising results obtained with cellulose, we applied heterogeneous catalysis to chitin and chitosan (Scheme 1), to obtain GlcN. Thus, SiO_2_ A and B, SiO_2_-Al_2_O_3_ and the corresponding copper supported materials were tested at first in chitosan depolymerisation.

**FIGURE 2:**
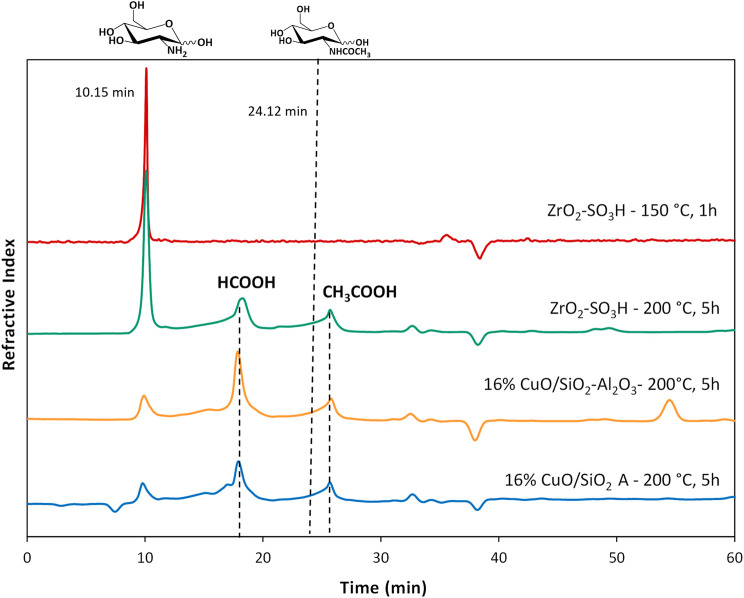
Chromatograms for chitosan depolymerisation over different catalysts (chitosan = 750 mg; cat = 400 mg; H_2_O = 50 mL, N_2_ = 4 atm).

The results are reported in Table 2, entries 1–6, together with the non-catalysed reaction. Surprisingly, the use of silicas and silica-alumina did not result in significant improvements in both conversion to soluble products (30%–38%) and GlcN yield (4%–7%), nor did the supported Cu catalysts, except for CuO/SiO_2_ B. CuO/SiO_2_ B gave a higher conversion (57%), but the GlcN selectivity was still low (5%). Different products were found in the HPLC chromatograms (Figure 2), but, due to the complexity of the reaction, we were able to identify only GlcN, and very small amounts of acetic and formic acid. To note that neither HMF or levulinic acid were found in the reaction mixtures. Future work will be devoted to the identification of added-value products, others than GlcN, even in small amounts.

**TABLE 2:** Chitosan depolymerization (T = 200°C; N_2_ = 4 atm; H_2_O = 50 mL; Chitosan = 750 mg; cat = 400 mg; t = 5 h).

Entry	Catalyst	C (%)	Y_GlcN_ (%)
1	no cat	27	4
2	SiO_2_-Al_2_O_3_	30	4
3	SiO_2_-ZrO_2_	32	5
4	16 CuO/SiO_2_ A	38	6
5	16 CuO/SiO_2_ B	57	5
6	16 CuO/SiO_2_-Al_2_O_3_	36	7
7	ZrO_2_	33	6
8	ZrO_2_-SO_3_H	32	23

One of the major issues in the depolymerisation of natural polymers (cellulose, chitin, and chitosan) is their robust crystallinity due the presence of extensive inter- and intramolecular hydrogen bonds. (Margoutidis et al., 2018) These exploratory experiments shed light on the more difficult depolymerization of chitosan with respect to what observed with cellulose, and the same pool of catalysts that successfully drove the selectivity in cellulose hydrolysis revealed to be ineffective in the present case.

**FIGURE 3:**
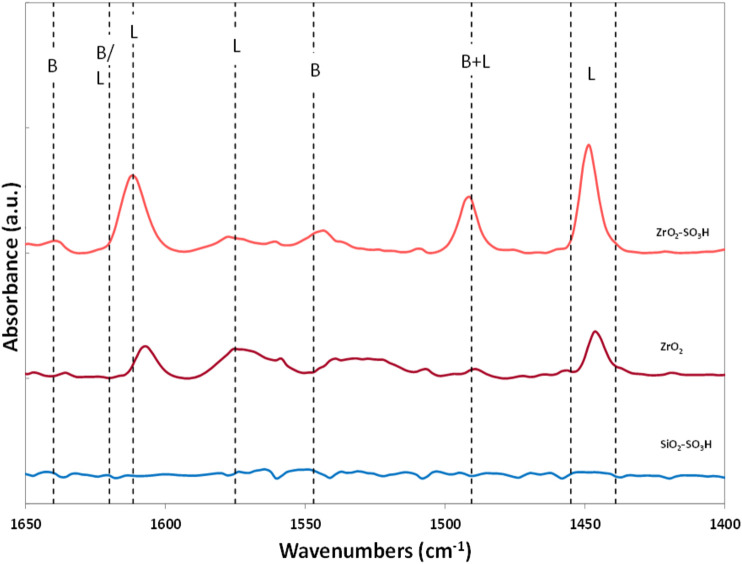
FT-IR spectra of pyridine on ZrO_2_ and ZrO_2_-SO_3_H.

Pursuing our effort in the search for an effective catalyst for the production of GlcN from chitosan, we therefore moved to sulfated zirconia (ZrO_2_-SO_3_H), known to possess strong Brønsted acidity. (Katada et al., 2000) The protocol used for the sulphation was straightforward (Fărcaşiu and Li, 1995), and the effectiveness of the treatment was confirmed by FT-IR of adsorbed pyridine (Figure 3). The parent ZrO_2_ shows only the presence of Lewis acid sites, with the typical bands at 1,446 cm^−1^ and 1,607 cm^−1^, while in the ZrO_2_-SO_3_H sample two signals at 1,542 cm^−1^ and 1,638 cm^−1^, ascribable to Brønsted acid sites, can also be detected. The sulfation does not seem to affect the structure of the oxides, as the surface area of the parent ZrO_2_ and the one of the ZrO_2_-SO_3_H materials do not show a significant difference (358 m^2^/g vs. 367 m^2^/g).

Interestingly, ZrO_2_-SO_3_H resulted to be an active material for chitosan depolymerisation (Table 2, entry 8). With this catalyst conversion reaches a value of 32%, coherent with results obtained on silica-based catalysts; however, GlcN selectivity is significantly higher, resulting in a 5-fold increase (23%). For comparison, the activity of the parent ZrO_2_ is similar to the one observed in the non-catalytic reaction, thus highlighting the fundamental role of the sulfation treatment.

ZrO_2_-SO_3_H is a promising catalyst for the selective hydrolysis of chitosan to GlcN. Differently to what observed with cellulose, where Lewis acid catalysts favour the monomer (glucose) product (Mariani et al., 2014), in chitosan depolymerisation the role of strong Brønsted acidity is critical to produce its building block (GlcN). In this respect, Omari et al. reported that Lewis acid homogeneous catalysts give higher HMF/LA yield, with respect to Brønsted ones, in the depolymerisation of chitosan by using SnCl_4_·5H_2_O and microwave irradiation. (Omari et al., 2012) Unfortunately, no information about the GlcN yields, that would be helpful to compare the effect of the different acidity, was reported.

**FIGURE 4:**
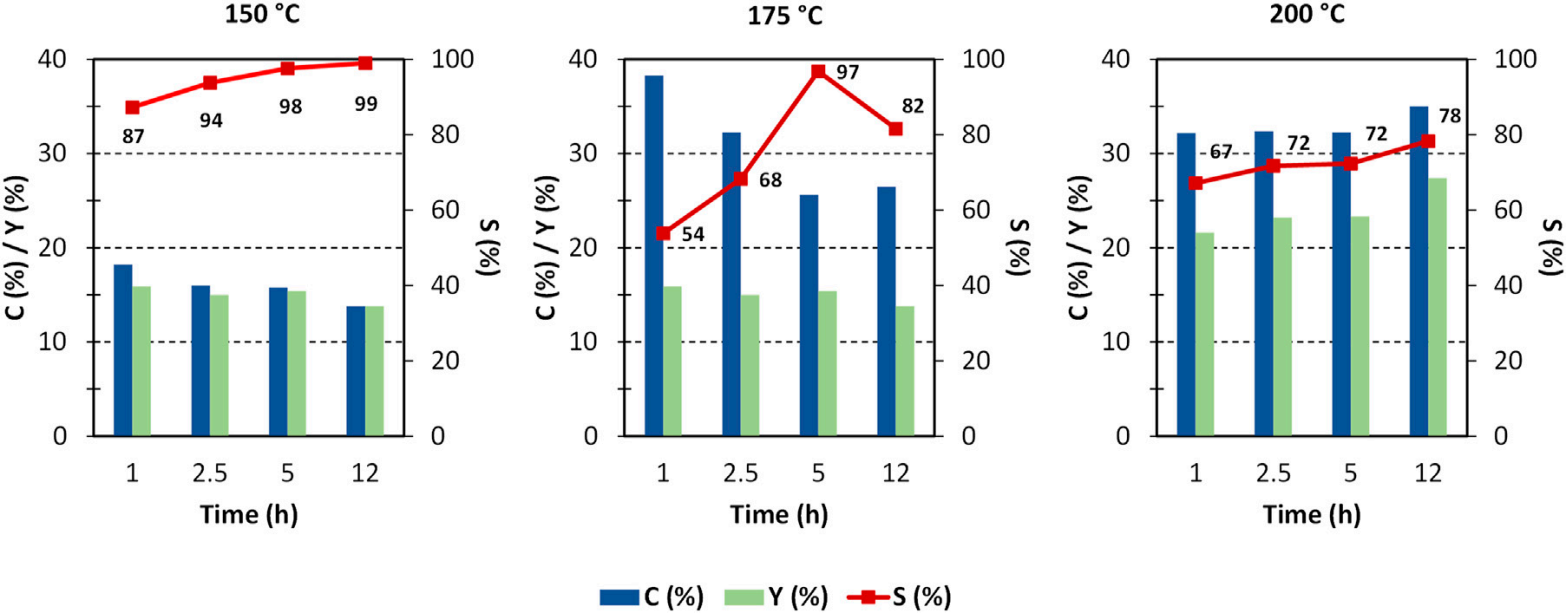
Chitosan depolymerisation over ZrO_2_-SO_3_H catalyst. C (%) = conversion to soluble products; Y (%) = GlcN yield; S (%) = GlcN selectivity, (chitosan = 750 mg; cat = 400 mg; H_2_O = 50 mL, N_2_ = 4 atm).

By virtue of the positive results shown by ZrO_2_-SO_3_H, we tried to improve the yield in GlcN by optimising the reaction parameters (time and temperature, Figure 4). The results show that most of chitosan converts within the first hour, with the formation of GlcN. A small increase in the GlcN yield was observed at 175°C and, especially, at 200°C, while conversion to a soluble product has a fluctuating behaviour. The maximum GlcN yield was 27%. The higher discrepancy between conversion and selectivity observed at 175°C could be related to the formation of soluble oligomers. It worth noting that the role of soluble oligomers in addressing the product distribution has already been put in light in cellulose deconstruction. (Chambon et al., 2011) A further increase to 200°C leads to easier hydrolysis of oligomers, but at the same time humins formation is favoured.

**FIGURE 5:**
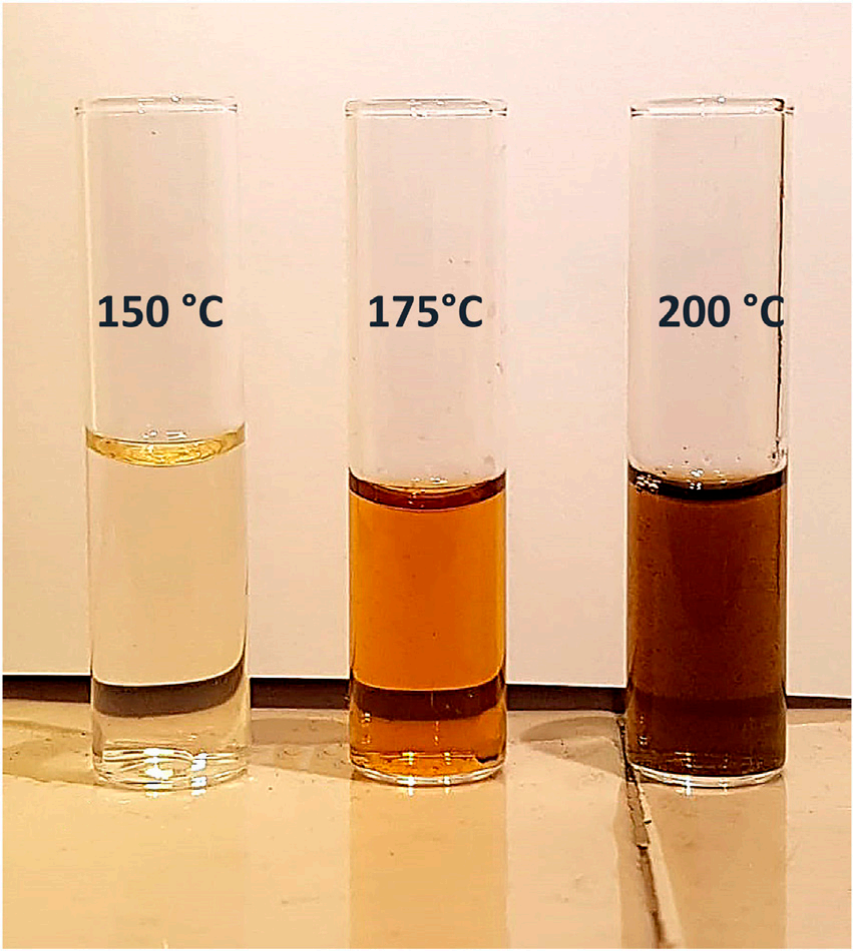
Filtered product solution collected at different temperature (chitosan = 750 mg; ZrO_2_-SO_3_H = 400 mg; H_2_O = 50 mL; N_2_ = 4 atm, t = 1 h).

This effect is clearly shown by observing the colour of the reaction solutions after filtration (Figure 5).

**FIGURE 6:**
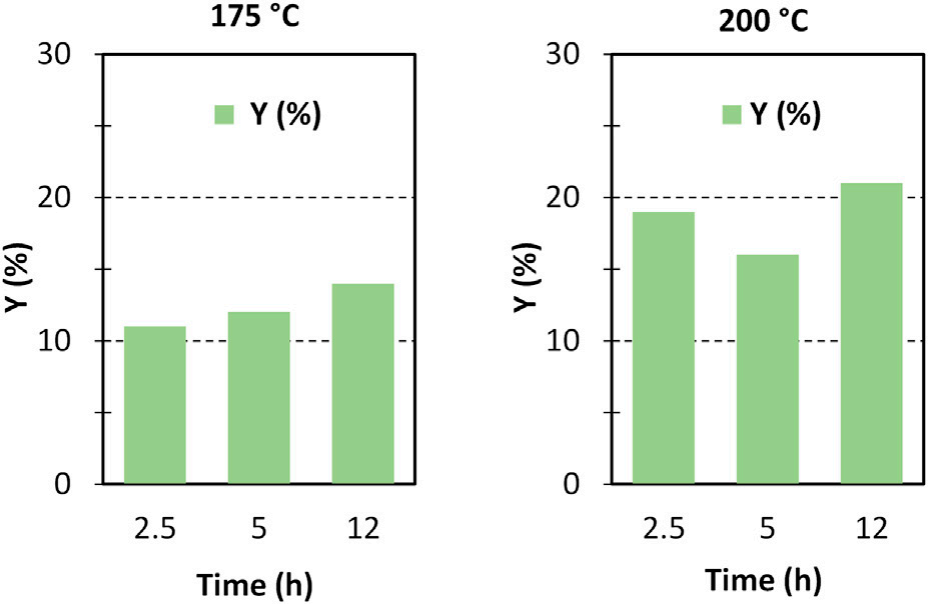
Chitin depolymerisation over ZrO_2_-SO_3_H catalyst. Y (%) = GlcN yield, (chitin = 750 mg; cat = 400 mg; H_2_O = 50 mL, N_2_ = 4 atm).

**SCHEME 2:**
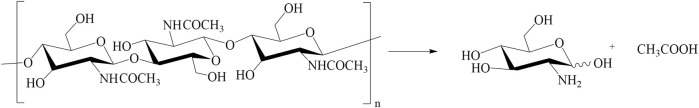
Chitin depolymerisation to GlcN.

**FIGURE 7:**
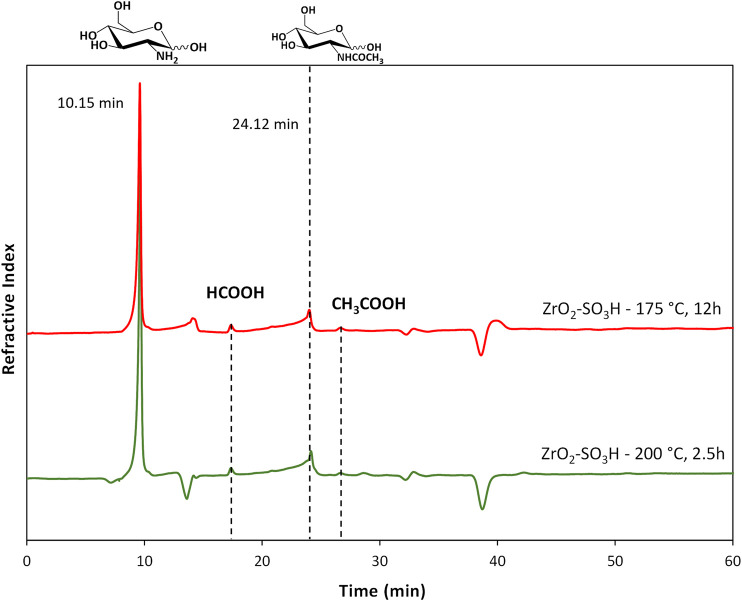
Chromatograms for chitin depolymerisation over ZrO_2_-SO_3_H (chitin = 750 mg; cat = 400 mg; H_2_O = 50 mL, N_2_ = 4 atm).

The formation of the decomposition products at higher temperature, likely due to the Maillard reaction (Wu et al., 2014), is highlighted by the browning of the solution going from 150°C to 200°C. In any case, the selectivity to GlcN is generally quite satisfying, with values that are generally higher than 65%–70%. As expected, the better selectivity was obtained at 150°C with a value of 87% at 1 h up to 99% after 12 h, and a single peak of GlcN was observed (Figure 2). With increasing temperature, the peak of GlcN is more intense, but at the same time the chromatogram reveals the formation of some by-products, such as formic acid and smaller amounts of acetic acid (Figure 2). In order to maximize the yield in GlcN the amount of the catalyst was increased up to 800 mg. This resulted in a GlcN yield of 44% at 200°C in 2.5 h. This preliminary result paves the way to further studies focused on the design of a more performant ZrO_2_-based material, in order to the decrease the catalyst amount and the reaction temperature (thus limiting humins formation). In particular, the development of an optimized sulfation protocol would lead to an increase of the Brønsted acid sites density on the catalyst surface and in turn to an improved activity. The catalytic depolymerization of chitin over the ZrO_2_-SO_3_H catalyst was also studied and the results are reported in Figure 6. The reaction produces directly GlcN as the main product (Scheme 2), while its acetylated form, the GlcNAc, was observed only in small amounts (Figure 7).

With respect to chitosan, the activity of the Zr-based catalyst decreases and a maximum GlcN yield of 21% (200°C, 12 h) was obtained.

By combining these results, with those obtained by some of us with cellulose, under similar experimental conditions, the following depolymerization tendency was observed: cellulose (Mariani et al., 2014)> chitosan > chitin. Copper-based catalysts and mixed oxides, such as SiO_2_-Al_2_O_3_ or SiO_2_-ZrO_2_, can be considered as promising materials for cellulose valorization, whilst fail with aminosaccharide polymers. With these substrates stronger acidity and sulfated systems are required. It has already been highlighted into the literature that the reactivity of chitin dramatically differs from those of cellulose and chitosan. (Deringer et al., 2016) (Yabushita et al., 2015) (Kaczmarek et al., 2019) Indeed, chitin has a higher crystallinity due to the presence of acetamido groups that enables the formation of numerous inter- and intra-molecular hydrogen bonds between linear chains.

Our results are very significant, if compared to those reported in the literature. Indeed, as above mentioned, the depolymerisation of chitin and chitosan are complicated and poorly studied reactions. Enzymatic routes are mainly pursued to obtain oligosaccharides or the deacetylation of chitin to chitosan. Moreover, they suffer from high cost and long reaction time, thus their use is often limited to the laboratory scale. (Kaczmarek et al., 2019) (Mohan et al., 2022) (Zhang et al., 2022) The use of toxic and dangerous homogeneous systems, such as mineral acids (HNO_3_, H_2_SO_4_, and HCl) or free radicals, sometimes with the aid of mechanical treatments, is reported so far. (Harish Prashanth and Tharanathan, 2007) (Einbu and Vårum, 2008) (Zhang and Yan, 2017) (Yabushita et al., 2015) Some processes end up in the loss of the N atom from the resulting product. This is the case of ionic liquids (Hou et al., 2020) and of a combination of SnCl_4_·5H_2_O with microwave irradiation (Omari et al., 2012), where HMF and levulinic acid were obtained. When a heterogeneous catalyst is used, its combination with a homogeneous one is always requested. For example, Amberlyts-15 and H_2_SO_4_ gave a GlcN yield of 58%. (Dai et al., 2019), while a glucose-derived solid acid catalyst resulted in a GlcN yield of 98.1%, but only after complete dissolution of chitosan in a hydrochloric acid solution. (Zhang et al., 2018) If the solid acid (H-Mordenite) is not assisted by a homogeneous one only oligomers were obtained, as results of an incomplete deconstruction of chitosan into the monomer. (Pandit et al., 2020) In this paper, we presented a new approach, in which the heterogeneous catalyst is used alone. The fruitful marriage between a high surface area zirconia and a simple sulfation protocol, gave us an active, selective, and easy-to-prepare material. Future work will be focused on the use of different zirconia-based materials (Zaccheria et al., 2020), and on the investigation of different sulfation procedures, to design a more performant system.

## Conclusion

A series of heterogeneous catalysts, based on oxides and mixed oxides have been studied in the reaction of chitosan and chitin depolymerization. They revealed to be much more recalcitrant to deconstruction with respect to cellulose. On the other hand, a material having strong Brønsted acidity, namely ZrO_2_-SO_3_H, resulted in a good selectivity towards GlcN and a yield of 21% from chitin and 44% from chitosan. To the best of our knowledge, our protocol is the first example relying on the use of a heterogeneous catalyst alone, active without the addition of any additives or mineral acids. This represents a step ahead, compared to homogeneous-catalysed processes that require massive neutralisation and purification steps, or the enzymatic ones that suffer for high costs and long reaction times. (Kaczmarek et al., 2019).

## Data Availability

The raw data supporting the conclusion of this article will be made available by the authors, without undue reservation.
